# A Case of Tropical Pulmonary Eosinophilia With Incomplete Response to Diethylcarbamazine Therapy

**DOI:** 10.7759/cureus.34359

**Published:** 2023-01-29

**Authors:** Ananda Datta, Pritam Chhotray, Banani Jena, Raghavendrun Sivasankar

**Affiliations:** 1 Pulmonary Medicine, Institute of Medical Sciences and SUM hospital, Bhubaneswar, IND

**Keywords:** filariasis, centrilobular nodules, diethylcarbamazine, tropical pulmonary eosinophilia, peripheral eosinophilia

## Abstract

Tropical pulmonary eosinophilia (TPE) is a specific pulmonary manifestation of lymphatic filariasis. There is overwhelming infiltration of eosinophils in the lung parenchyma in response to microfilaria. The characteristic features include paroxysmal respiratory symptoms, strikingly high blood eosinophil count, elevated level of immunoglobulin (Ig) E along with high titer of anti-filarial antibody. Treatment with diethylcarbamazine (DEC) has an excellent favorable response. However, recovery may often be incomplete.

We present a case of a 36-year-old man with TPE who had complete symptomatic improvement after a three-week course of DEC, but only a partial response in radiological and pulmonary function abnormalities.

## Introduction

Tropical pulmonary eosinophilia (TPE) is the pulmonary manifestation of a hyperresponsive immunological reaction to the infection caused by filarial parasites. The majority of cases are clustered in the tropical and subtropical regions of the world where filariasis is endemic [1]. The condition is usually characterized by dry cough, wheezing, and marked peripheral eosinophilia. The radiological features include ill-defined nodules and interstitial infiltrates. Spirometry is usually suggestive of a mixed restrictive and obstructive pattern. The response to diethylcarbamazine (DEC) therapy is excellent with significant resolution of clinical features and peripheral eosinophilia. Often the radiographic and lung function abnormalities may persist even after the completion of treatment [2]. Here, we present a case of TPE who had an incomplete radiologic and pulmonary functional response after DEC therapy.

## Case presentation

A 36-year-old male patient presented with complaints of dry cough and shortness of breath on exertion for two months. His cough was worse at night. He also complained of low-grade intermittent fever for two months. His appetite was normal and he denied any history of weight loss, joint pain, night sweats, chills, rash, rhinorrhea, epistaxis, hemoptysis, hematuria or burning sensation in hands and feet. There was no history of smoking or any kind of drug intake. His past medical history was unremarkable. He was a resident of a village in the eastern part of India and worked as a manager on a poultry farm.

**Figure 1:**
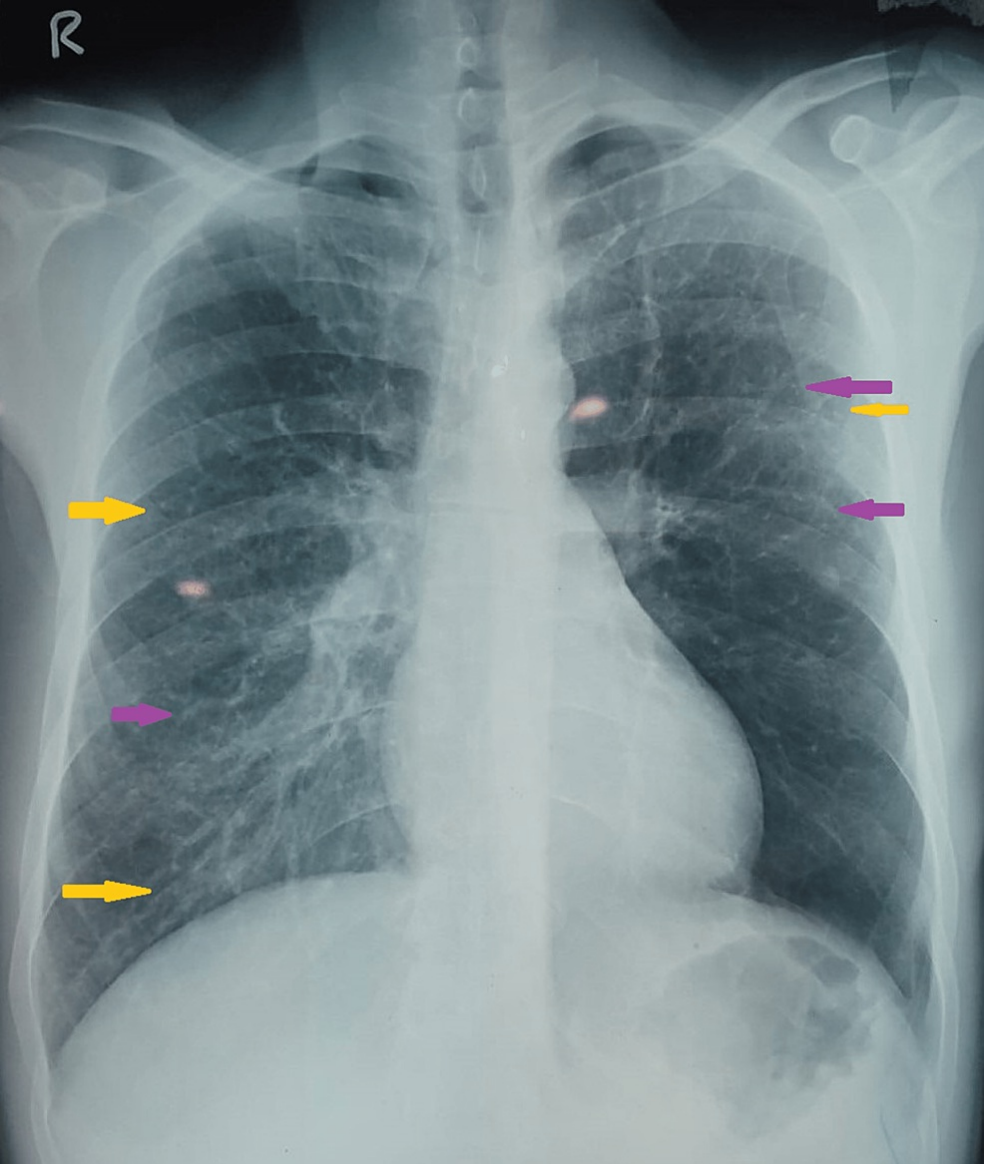
Chest radiograph showing reticulonodular opacities involving all zones of both lung fields Purple arrows indicate reticular opacities. Yellow arrows indicate nodular opacities.

Two weeks before presenting to our medical care facility, he was diagnosed with a lower respiratory tract infection by a private practitioner and was prescribed a short course of oral antibiotics. But he continued to have fever, cough, and breathlessness. On presentation to our facility, his physical examination was notable for rhonchi throughout both lung fields. His vital signs were normal. The results of initial laboratory investigations were as follows: hemoglobin 12.8 gm/dL (reference range: 13-17 gm/dL), total leukocyte count 50,230/µL (reference range: 4,000-11,000/µL), absolute eosinophil count 37,510/µL (reference range: 40-440/µL), platelet count 151,000/µL (reference range: 150,000-400,000/µL), and random blood sugar 93 mg/dL (reference range: 70-100 mg/dL). Peripheral blood smear showed leukocytosis with marked eosinophilia without any left shift. Liver function tests and kidney function tests were within normal range. The chest radiograph showed the presence of reticulonodular opacities involving all zones of both lung fields (Figure 1).

**Figure 2:**
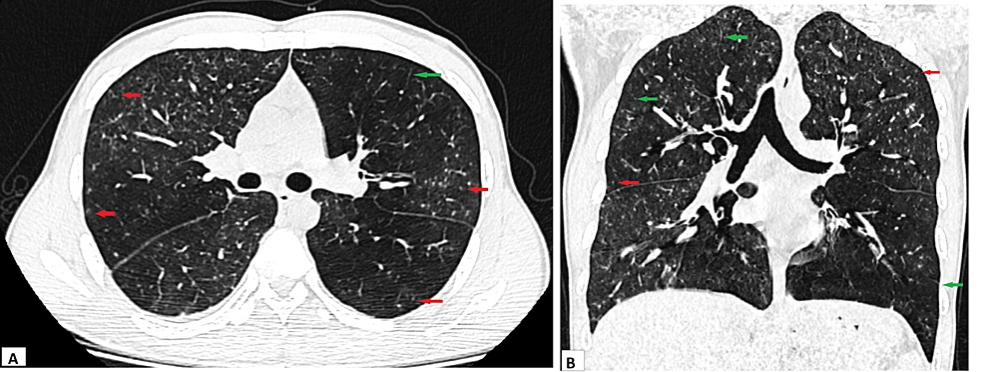
HRCT scan of thorax axial view (A) and coronal view (B) showing diffuse centrilobular nodules (red arrows) and smooth interlobular septal thickening (green arrows) along with mosaic attenuation in the bilateral lung parenchyma. HRCT: High-resolution computed tomography Red arrows indicate centrilobular nodules. Green arrows indicate smooth interlobular septal thickening.

High-resolution computed tomography (HRCT) scan of the thorax revealed the presence of mosaic attenuation in the bilateral lung parenchyma. There were multiple centrilobular nodules along with smooth interlobular septal thickening involving all the lobes bilaterally (Figure 2).

Cytological analysis of bronchoalveolar lavage fluid (BAL) showed marked eosinophilia. Smear examination and cultures of BAL fluid revealed no pathogenic microorganism and GeneXpert MTB/RIF did not detect *Mycobacterium tuberculosis*. Spirometry was suggestive of combined obstructive and restrictive changes.

The Mantoux test was negative. The stool examination did not show any parasites. Urine examination did not reveal any protein, red blood cells, or sediment. Serum immunoglobulin (Ig) E level was 9915 U/L (reference range: less than 150 U/L). Serum *Aspergillus fumigatus*-specific IgE level was 0.08 kU/L (reference range: less than 0.35 kU/L), while serum *A. fumigatus*-specific IgG level was 13 mg/L (reference range: less than 27 mg/L). Antineutrophil cytoplasmic antibodies were negative. Anti-filarial antibody was positive by the immunochromatographic method. 

The patient was started on DEC therapy at a dose of 100 mg thrice daily. Cough improved within three days of treatment initiation and the fever subsided after five days. Subsequent serial blood counts showed a decrease in eosinophil count (Table 1).

**Table 1:** Trend of total leukocyte count and absolute eosinophil count during the course of treatment DEC: Diethylcarbamazine

Parameters	On admission	After 5 days of starting DEC	After 10 days of starting DEC	At 7 days after completion of the DEC course
Total leucocyte count, per µL (Reference range: 4,000-11,000/µL)	50,230	20,390	13,200	9,000
Absolute eosinophil count, per µL (Reference range: 40-440/µL)	37,510	7,000	5,590	591

**Figure 3:**
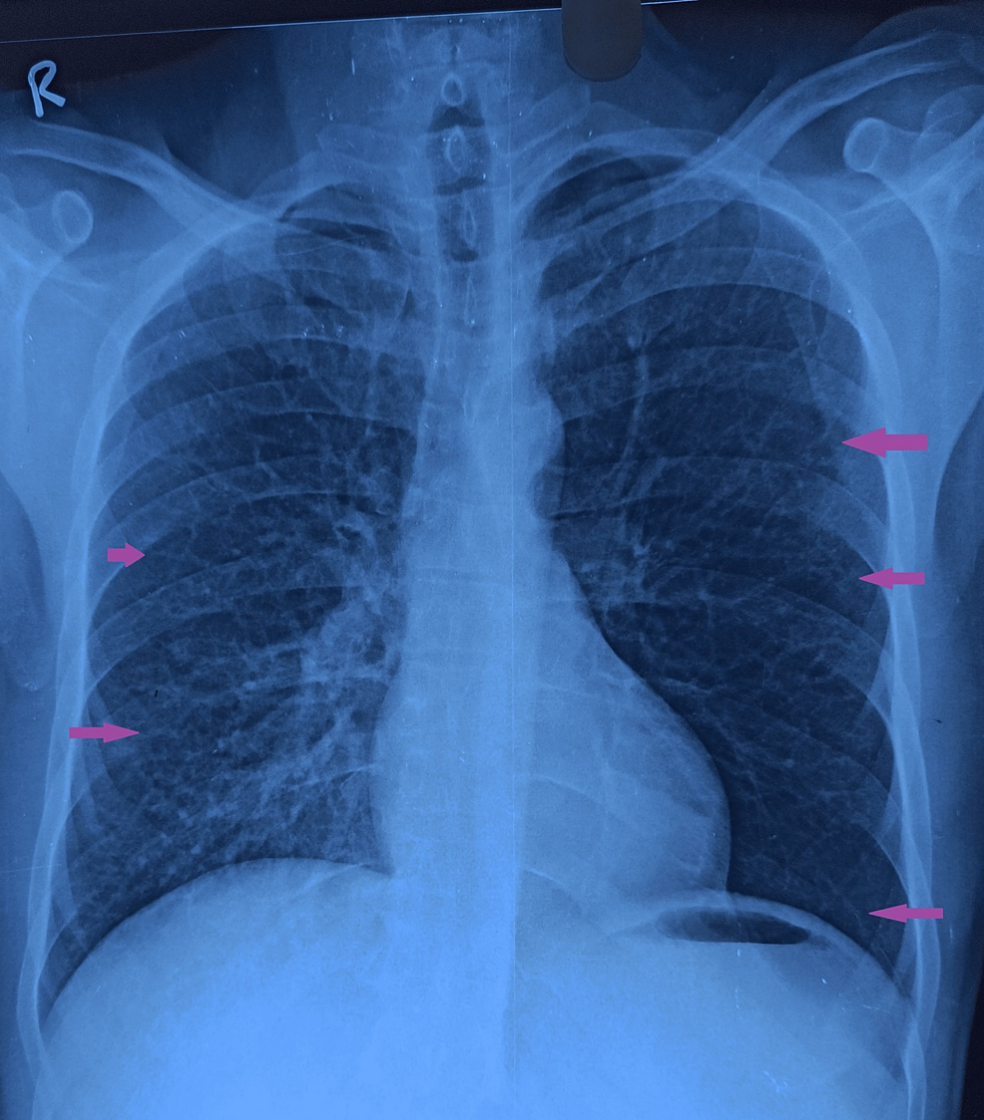
Follow-up chest X-ray showing persistence of reticular opacities in bilateral lung fields Purple arrows indicate reticular opacities

Diethylcarbamazine was administered for three weeks. The patient became completely asymptomatic. After four weeks of completion of the DEC course, the chest radiograph and spirometry were repeated. Chest X-ray showed the persistence of reticular opacities bilaterally (Figure 3).

Also, follow-up spirometry showed partial improvement in lung function with the persistence of restrictive abnormality (Table 2).

**Table 2:** Spirometric values before and after treatment FVC: Forced vital capacity, FEV1: Forced expiratory volume in the first second

Spirometric parameters	Before treatment	After treatment
FVC	2.32 (71%)	2.45 (75%)
FEV1	1.50 (55%)	1.99 (73%)
FEV1/FVC	64%	81%

## Discussion

Pulmonary eosinophilia is a heterogeneous group of infectious and non-infectious conditions that involve infiltration of eosinophils into the lung parenchyma and the airways. It is usually associated with peripheral eosinophilia. Tropical pulmonary eosinophilia is a distinct eosinophilic pulmonary process of lymphatic filariasis occurring as an immunological response to microfilaria trapped in the lungs. Lymphatic filariasis occurs due to infections with mosquito-borne nematodes of *Wuchereria bancrofti*,* Brugia malayi*,and* Brugia timori* [3]. As the name suggests, it is commonly seen in tropical areas like the Indian subcontinent, South East Asia, South America, Africa, parts of China, and South Pacific islands. However, many cases have been reported from non-endemic parts of the world that have been attributed to the history of travel to the endemic regions [1].

Less than 1% of patients with lymphatic filariasis have pulmonary eosinophilia. The affected individuals are commonly older children and young adults with male preponderance. Approximately 7% of cases of TPE show extrapulmonary manifestations [4]. After the establishment of infection in humans, the mature filarial parasites inhabit lymphatics. They release microfilariae periodically into lymphatic vessels and the bloodstream. During dissemination, microfilariae get trapped in the pulmonary circulation and later disintegrate to release various antigens. This results in an intense eosinophilic inflammatory response in the lung parenchyma. Eosinophils are the primary immune cells involved in microfilariae clearance. Tissue damage is caused by the release of the contents of eosinophilic granules. The antigens often can reach systemic circulation leading to the involvement of various extrapulmonary organ systems like the reticuloendothelial system, gastrointestinal system, and rarely musculoskeletal system [2,5].

The onset of symptoms is usually gradual. The respiratory symptomatology includes non-productive cough, episodic breathlessness, wheezing, and chest pain. The cough is frequently paroxysmal and nocturnal. If expectoration is present, it is scanty and viscid. Systemic features include fever, malaise, fatigue, and weight loss. Respiratory system examination may reveal rhonchi and basal crepitations. Peripheral lymphadenopathy and hepatosplenomegaly may be present [2].

The characteristic laboratory finding is an elevated absolute eosinophil count that is usually more than 3,000/µL. The count may be as high as 80,000/µL. Serum IgE level is elevated above 1,000 U/L. Microfilariae are usually undetectable in peripheral blood smear examination [1]. The filaria-specific antibodies can be detected in blood by immunochromatographic and enzyme-linked immunosorbent assay using recombinant antigens of the filarial parasite [6,7].

Chest radiographs may be normal in one-fifth of the cases. The common radiographic abnormality is reticulonodular lesions in bilateral mid and lower zones. Small ill-defined nodules up to 3 mm in diameter can appear as miliary mottling. The CT is superior in the delineation of radiologic abnormalities. It can show ill-defined broncho-centric nodules, ground-glass infiltrates, and smooth interlobular septal thickening. Uncommon findings are bronchiectasis, air trapping, mediastinal lymphadenopathy, and pleural effusion [5,8].

Spirometry usually shows a restrictive pattern along with a mild to moderate degree of obstruction. The diffusion capacity of the lung for carbon monoxide is also reduced. Mild hypoxemia can also be present in acutely ill patients [1]. Bronchoalveolar lavage fluid analysis indicates an intense eosinophilic response in the lungs [9].

In tropical regions, helminthic infestation is the most frequent cause of pulmonary and peripheral eosinophilia. Important pathogens are roundworm, hookworm, Toxocara, and Strongyloides. The clinical presentation of these helminthic infections is often quite similar to TPE [10]. Non-infectious causes of pulmonary eosinophilia include allergic bronchopulmonary aspergillosis, bronchial asthma, acute eosinophilic pneumonia, chronic eosinophilic pneumonia, idiopathic hypereosinophilic syndrome, eosinophilic granulomatosis with polyangiitis, granulomatosis with polyangiitis, and drug-induced hypersensitivity reaction [3,11]. The above-mentioned differential diagnosis should be considered during the evaluation of peripheral eosinophilia with pulmonary infiltrates. The diagnostic criteria for TPE include (a) history of residence or travel to a filarial endemic region; (b) paroxysmal and nocturnal cough with dyspnea; (c) leukocytosis with peripheral blood eosinophilia, usually above 3,000/µL; (d) elevated serum IgE, above 1,000 U/L (e) elevated filarial antibody titer; (f) pulmonary infiltrations in radiology; and (g) clinical improvement with DEC [2].

Diethylcarbamazine has been the drug of choice for lymphatic filariasis for more than 50 years. Diethylcarbamazine therapy results in a dramatic improvement in signs and symptoms in most cases of TPE. The standard treatment dose is oral DEC 6 mg/kg per day in two to three divided doses for three weeks. Most patients show marked symptomatic, functional, and radiographic improvement after three weeks [2]. The analysis of a case series showed that response in laboratory and lung function parameters following treatment was variable [12]. An incomplete response results from persistent low-grade inflammation that leads to interstitial fibrosis and chronic respiratory insufficiency. Almost 5% of the cases may remain unresponsive to DEC. Delay in the administration of DEC therapy can result in progressive interstitial fibrosis and irreversible impairment of lung function [1,2]. There is no correlation between symptom duration prior to diagnosis and the level of eosinophilia with radiographic or functional changes. Post-treatment radiological and functional changes may persist even in an illness of a short duration [13,14,15]. However, DEC may be ineffective in about 20% to 40% of cases with chronic symptoms.

Although steroid has been shown to suppress persisting inflammation, there is no consensus about the dose and duration of use of corticosteroid [2]. In addition, restrictive pulmonary changes have been seen to persist even after the use of long-term steroids despite improvement in clinical and eosinophil count [13,16]. The rate of relapse after DEC therapy is estimated to be 20% over a follow-up period of five years [2].

## Conclusions

In our case of TPE, the patient responded partially to three weeks of DEC therapy. Radiologic and functional abnormalities persisted even after four weeks of treatment completion. Tropical pulmonary eosinophilia is one of the pulmonary eosinophilic syndromes of known etiology. Hypersensitivity reaction induced by microfilarial antigen results in pulmonary as well as peripheral eosinophilia. The disorder can be diagnosed with the help of the diagnostic criteria consisting of demographic, clinical, radiological, and hematological features, and response to DEC treatment. Although DEC is effective for the resolution of respiratory symptoms, radiologic and spirometry findings may persist in 4% to 5 % of cases. 
